# Treating *Mycoplasma genitalium* (in pregnancy): a social and reproductive justice concern

**DOI:** 10.1007/s40592-024-00200-7

**Published:** 2024-07-11

**Authors:** Ulla McKnight, Bobbie Farsides, Suneeta Soni, Catherine Will

**Affiliations:** 1 The Sociology and Criminology Department, University of Sussex, Freeman Building G30, Falmer Brighton, BN1 9QE UK; 2 Brighton and Sussex Medical School, Falmer, UK; 3 Consultant in Sexual Health and HIV, University Hospitals Sussex, Honorary Clinical Senior Lecturer, Brighton and Sussex Medical School, Falmer, UK

**Keywords:** Antimicrobial stewardship, Consequentialist ethical evaluation, Sexual and reproductive health care, Reproductive justice

## Abstract

Antimicrobial Resistance is a threat to individual and to population health and to future generations, requiring “collective sacrifices” in order to preserve antibiotic efficacy. ‘Who *should* make the sacrifices?’ and ‘Who *will* most likely make them?’ are ethical concerns posited as potentially manageable through Antimicrobial Stewardship. Antimicrobial stewardship almost inevitably involves a form of clinical cost-benefit analysis that assesses the possible effects of antibiotics to treat a diagnosed infection in a particular patient. However, this process rarely accounts properly for patients – above and beyond assessments of potential (non)compliance or adherence to care regimes. Drawing on a vignette of a pregnant woman of colour and migrant diagnosed with Mycoplasma genitalium, a sexually transmissible bacterium, this article draws out some of the ethical, speculative, and practical tensions and complexities involved in Antimicrobial Stewardship. We argue that patients also engage in a form of cost-benefit analysis influenced by experiences of reproductive and social (in)justice and comprising speculative variables - to anticipate future possibilities. These processes have the potential to have effects above and beyond the specific infection antimicrobial stewardship was activated to address. We contend that efforts to practice and research antimicrobial stewardship should accommodate and incorporate these variables and acknowledge the structures they emerge with(in), even if their components remain unknown. This would involve recognising that antimicrobial stewardship is intricately connected to other social justice issues such as immigration policy, economic justice, access to appropriate medical care, racism, etc.

## Introduction

I was like very, very concerned that they could give me something that could harm the baby, even though they knew I was pregnant. But I was like, I don’t know, it’s like medicine, I don’t know if, at the end of the day it’s still drugs. So I don’t know if could be something affecting the baby. But she was like very, no, we have a good team here, they’re going to look into this. We’re going to wait to see if you are positive and then we’re going to do something.

But she didn’t jump. She just say, yeah, stay calm, enjoy positive test of pregnancy, and she was very, very, very good. (Interview with Antonia)

I was kind of, okay, if I need to be treated for something let’s do it. At least they found something and then this is the right treatment. It was just like, let’s get done, let’s take it. I don’t know, I really don’t know if could get something serious like if I didn’t take the…I didn’t think about that. I was just like, okay, I have something, I need…I really need to clean my body from this, so let’s just take the medicine (Interview with Antonia).

Antimicrobial stewardship is a complex issue, but amongst other elements, it almost inevitably involves a form of clinical cost-benefit analysis (CBA) that assesses the possible effects of the use of antibiotics to treat a diagnosed infection in a particular patient. However, this process rarely accounts properly for patients – above and beyond assessments of potential (non)compliance or adherence to care regimes (GOV.UK, 2023). Drawing on an anonymised vignette of a pregnant woman of colour and migrant, whom we refer to as Antonia, diagnosed with *Mycoplasma genitalium* (*M. genitalium*) caused by a sexually transmissible bacterium, this article draws out some of the ethical, speculative, and practical tensions and complexities involved in antimicrobial stewardship when being treated for and *M. genitalium* infection in an English National Health Service (NHS) sexual health clinic. We suggest that patients also engage in a form of cost-benefit analysis comprising speculative variables - influenced by experiences of reproductive and social (in)justice - to anticipate future possibilities (Eaton and Stephens 2020; SisterSong 2018; Luna 2009; Zavella 2016). These processes have the potential to have effects above and beyond the specific infection AMS was activated to address. We contend that efforts to practice and research AMS should accommodate and incorporate these variables and acknowledge the structures they emerge with(in), even if their components remain unknown. This would involve recognising that AMS is intricately connected to other social justice issues such as immigration policy, economic justice, access to appropriate medical care, racism, etc. (Price 2020: 341).

First identified in the early 1980s (Tully et al., 1981), M. *genitalium* is a sexually transmissible pathogen that has been associated with post-coital bleeding, cervicitis and pelvic inflammatory disease in cisgender women^2^ (Soni et al., 2019: 940). Further to this, *M. genitalium* has been linked with endometritis (Wood et al., 2021: 1), pre-term birth and spontaneous abortions (Jensen and Bradshaw 2015a: 1; Soni et al., 2019: 940), miscarriages (Donders et al., 2017: 505), preterm rupture of membranes and low birth rate (Vallely et al., 2018: 2). Despite this alarming list, there is a paucity of data about the risks of *M. genitalium* in cisgender women/non-binary people and/or transmen with uteruses in relation to their reproductive health, given the long-standing precautionary approach to involving pregnant people in medical research. What is known is that the microbe is exceptionally proficient at developing resistance to new treatment methods and is consequently infamous for being difficult to treat (Conway et al., 2020: 197; Jensen and Bradshaw 2015b: 7). Furthermore, it is also known that *M. genitalium* infections are often symptomless and may clear without antibiotics. Thus, over-testing and treating may unnecessarily further increase antimicrobial resistance (AMR). There is, therefore, concern within the field of sexual health in England that an increased public awareness and focus on *M. genitalium* will only increase demand for unnecessary testing and treating of infections. Moreover, the limited availability of resistance testing within the NHS increases pressure not unnecessarily to test for *M. genitalium*. Accordingly, *M. genitalium* has become the *Cassandra bacterium* of AMR in that it prophesies a post-antibiotic era. It also provides an empirical example that explicates debates about social and reproductive justice, the ethicality of antimicrobial stewardship, and efforts to reduce AMR. The article is structured as follows: a brief discussion of the threat of AMR and some of the contingencies of antimicrobial stewardship will precede a discussion of our research methods, followed by Antonia’s vignette, reflections, and final thoughts.

### The threat of antimicrobial resistance

Antimicrobial resistance (AMR) is considered a substantial threat to individual and population health and future generations (Streicher 2021: 293; Murray et al., 2022), requiring “collective sacrifices” to preserve antibiotic efficacy for the future (Littmann and Viens 2015: 210-1). Failure to prevent AMR is understood as leading to a post-antibiotic era where strains of infections emerge and spread, becoming resistant to existing antimicrobials as bacteria change in response to antibiotic exposure (Giubilini 2017: 653; Will 2020). With limited or no effective antibiotics to treat bacterial infections (Irwin 2020: 1), medical procedures we have come to see as routine, such as chemotherapy and major surgery (GOV.UK 2022) and curative treatments for sexually transmitted infections, are at serious risk (Fernández-Huerta et al., 2020; Unemo and Jensen 2017).

An additional complexity arises as a result of the unequal global distribution of biomedical medicines and appropriate care, which has, as a consequence, that lack of access to antimicrobials significantly drives drug resistance (Hellamand et al., 2022; Murray et al., 2022), with low-income settings being disproportionately affected and at risk (Hellamand et al., 2022; Murray et al., 2022). Thus, in the interests of fairness, equality and efforts to reduce AMR - excessive and inappropriate use of antibiotics - must be reduced in some global locations, and appropriate use of the same agents needs to increase in others (Littmann and Viens 2015; Hays et al., 2022). Therefore, at a global level, AMR raises important ethical and moral questions pertaining to social and reproductive justice (Ross and Solinger 2017b; Eaton and Stephens 2020). These questions concern the disproportionate effects of AMR on poorer countries (Broom et al., 2019: 217), ethnic and racial minorities (Abdul-Mutakabbir and Simiyu 2022) and marginalised populations (Adebisi and Ogunkola 2023), and what a fair distribution of remaining antimicrobials would be. Furthermore, these questions also concern the disproportionate maternal morbidity and mortality rates for women of colour in the UK (Knight et al., 2020; Knight et al., 2021) and how ambiguous legal status exacerbates the inequalities faced by racialised and ethnic minority women (Pellegrino et al., 2021; Qureshi et al., 2020; Prabhat 2020). Moreover, and of particular significance to this article is the related issue of how large-scale drug-based studies have routinely excluded ‘women of childbearing age’ irrespective of their individual reproductive history or current position in relation to contraception, etc. (Yakerson 2019; Ravindran et al., 2020).

AMR is most often discussed at a societal or even global level. Still, at the outset, we identify as a moral imperative the need to protect the interests of individual patients in the context of a worldwide endeavour to limit the use of antibiotics. It is also important to understand that decisions regarding AMR can get entangled with other morally significant issues, some of which are equally urgent. Given the details of our vignette, we are especially keen to stress the rights of pregnant people - particularly when they are racialised (migrants) - too many of whom are and have historically been disadvantaged in the context of cost-benefit and risk analysis relating to (access to) research and sexual and reproductive healthcare (Knight et al., 2021; Roberts 1993; Ismaili M’hamdi and de Beaufort 2021) including abortion care (McGuinness and Montgomery 2020; Goodwin 2020). Whilst we might all, as citizens, be required to make sacrifices in the interests of promoting public health goals, we argue that this is not analogous to a pregnant person being required to make health-related sacrifices to protect a foetus, and these issues should not become confused or conflated. When faced with a medical decision relating to their health, moral and clinical agency rests with the pregnant person, who should be assisted in deciding how *they* wish to proceed and how *they* hope to balance personal, societal, and foetus-related considerations. An effective and lawful treatment option should not be withheld or withdrawn from a pregnant person without their knowledge and consent. In supporting a pregnant person’s decision-making and choice, healthcare providers must remain open, supportive and non-directive, even if it emerges that they would place a different moral emphasis on the decision (Roberts 1999). It is also important to acknowledge that approaches to AMR often assume that sacrifices should be made now for the benefit of unknown others and/or future generations, which is a politically, if not ethically, contestable issue (Littmann and Viens 2015: 211; Will 2020). ‘Who *should* make the sacrifices?’ and ‘Who *will* most likely make them?’ are ethical concerns posited as potentially manageable through antimicrobial stewardship.

### Managing AMR with antimicrobial stewardship

In response to the threat of AMR and at a macro level, antimicrobial stewardship (from here on referred to as stewardship) is imagined as having the potential to avert a post-antibiotic era by preserving antibiotic efficacy through a systematic “…approach to promoting and monitoring judicious use of antimicrobials” (NICE 2022a), and at a national level “… may well determine to a large degree the future scope, utility, and effectiveness of the English NHS” (Dixon and Duncan 2014: 150). At its core, stewardship involves providing optimal care to patients in need of antibiotics while also ensuring that the chosen treatment pathway is the least likely to enable microbes to develop or obtain resistance (Doron and Davidson 2011: 1114; UKHSA 2021: 116). As such, stewardship is intended to benefit individuals *and* society – now and in the future (Dixon and Duncan 2014: 145). However, who decides what this might look like in practice and what is needed to enact or prevent these speculative futures is another ethical concern that requires more attention.

Thus, at a micro level, within sexual health clinics, healthcare providers are tasked with the responsibility of prescribing for individual patients. They are ethically required to focus on the interests of individual patients while supporting more socially oriented goals. These obligations may, at times, contradict. Moreover, fulfilling these responsibilities in light of budget and institutional restrictions (Bringedal et al., 2018: 242) and the expectations of patients is not straightforward. Enacting these obligations can involve undertaking a form of Cost-Benefit Analysis (CBA) using speculative variables to enhance imagined futures and the lives of humans and non-humans – e.g., people, human non-persons, and microbes - that might inhabit them. In practice, this requires healthcare providers to imagine the potentiality of the patient, her behaviour, the treatment she is offered and the progression of the microbe within her body and as the microbe may spread through her sexual networks and potentially spread vertically to any children she may have. This is in addition to speculating on these potentialities’ impact on AMR and efforts to prevent post-antibiotic futures.

Given AMR’s public health focus, the objective of a CBA is to determine which speculative alternatives would provide the most significant rewards for society (Choy 2018: 1). The ultimate and, therefore, the “morally most desirable” possibility would be the prospect that would positively enhance, to the highest degree, both society and all individuals (Choy 2018: 1). In short, the assessment process dictates that when faced with a choice as to whether or not to do X, one must consider if the benefits of X would be more significant than the next best option. If they are, X should be the chosen option. The results would garner the greatest possible benefits if this process is used to assess all choices (Layard and Glaister 1994: 1). This process is like a consequentialist style ethical evaluation, balancing considerations of harm and benefit in the context of scientific and moral uncertainty, imagined futures and institutional restrictions, where the individual patient’s interests must be considered but not overcounted. Significantly, in this context, the process is complicated because the variables that could become apparent - in the future - and tip the balance between harm and benefit are known to be unknowable in real time. In other words, the process involves a place marking for the influence of the unknown.

Decisions based on a consequentialist approach must allow for the possibility that hoped-for benefits are not secure. In practice, the harms one wishes to avoid might be greater or lesser than one predicts. In most situations, we have data to rely on to help us feel a bit more confident in our analysis, but in the treatment of pregnant people, our data sets are almost guaranteed to be limited. This is due to the absenting of pregnant people from medical research and the fact that clinical data will reflect the social and moral pressures (and, in some domains, legal and financial strictures) placed upon pregnant people when they consider treatment decisions which could or will have an impact upon the foetus they are carrying – and increasingly the foetus that they could hypothetically carry in the future (Goodwin 2020). For many years, almost without comment, large-scale drug-based studies have excluded ‘women of childbearing age’ irrespective of their individual reproductive history or current position in relation to contraception, etc. (Yakerson 2019; Ravindran et al., 2020).

The epidemiological history of *M. genitalium* and the post-antibiotic future that *M. genitalium* prophesies, in addition to medical care systems, the patient and their familial and sexual networks and histories that are outside of the NHS’s purview, further obfuscate a CBA analysis by adding additional unknowable variables into the equation. In other words, what is known about *M. genitalium* and the person being cared for (e.g., the prevalence of macrolide resistance mediating mutations and the resistance profile of the patient’s *M. genitalium*) and what is speculated about them (e.g., the effect of treating the patient’s infection will have on AMR, or a foetus and the likelihood of the patient adhering to treatment plans or procuring antibiotics elsewhere) could all take part in a CBA. In other words, X and the next best option are made up of both knowable (insofar as medical facts come to matter and be knowable) (Moser 2008) and *unknown*, that is, speculative variables. We contend that adopting a reproductive justice framework could better anticipate and address the speculative variables and their potential effects.

### AMS as a social and reproductive justice concern

Reproductive justice had its impetus in the early 1990s when it was coined by a small group of Black and African American women in the USA (Daniel 2021: 1–2) in response to the pro-choice vs. pro-life binary and its failure to address the needs of women of colour (Ross 2022). Reproductive justice centres human rights and social justice (Eaton and Stephens 2020) and is simultaneously “a theory, a framework, and a praxis” — a distinct invitation to reflect upon and act within the world to facilitate societal and personal transformation (Daniel 2021: 1–2). Drawing on human rights and social justice (Eaton and Stephens 2020) reproductive justice moves away from notions of ‘choice’ as the ability to choose is not a privilege that most women of colour have access to (Eaton and Stephens 2020; SisterSong 2018; Luna 2009; Zavella 2016). Instead:

The reproductive justice framework recognizes the importance of linking reproductive health and rights to other social justice issues such as poverty, economic injustice, welfare reform, housing, prisoners’ rights, environmental justice, immigration policy, drug policies, and violence (Price 2020: 341).

While it was devised in a US-specific context, reproductive justice is global in scope and has the potential to enact change. The framework’s tenants pertain to three main areas: the right to have, the right not to have children, and the right to raise families in safe environments (Daniel 2021: 1–2). Integral to these tenants are women’s ability to freely exercise these rights without coercion (Price 2020: 341; Ross 2022) and the acknowledgement of the systematic practice of oppression women face using an intersectional lens (Price 2020: 341). We will illustrate how AMS is a reproductive justice concern.

## Methods

This article draws on ethnographic explorations of marginalisation and efforts to reduce antimicrobial resistance within sexual health clinics within four NHS Trusts in England. The research is part of a larger Wellcome Trust-funded project (214954/Z/18/Z), which focused on health inequalities and the organisms central to the current crisis in AMR. The research was originally planned to be an in-person ethnography that would take place within two different NHS trusts over the course of a few months with a comparative leg in the United States. However, a week after commencing fieldwork, COVID-19 imposed restrictions disrupted our plans. Eventually, after numerous hurdles, the project was reimagined as primarily a virtual ethnography, with data collection taking place from March 2020 to March 2022, with many unavoidable and lengthy stops and re-starts. The original ethnography was planned by Professor Catherine Will and Dr Ulla McKnight, but Professor Bobbie Farsides took over from Catherine as Principal Investigator in the early spring of 2021.

Staff employed at clinical sites offering sexual health services within the four participating NHS trusts were invited to participate in the research. Clinical collaborators within each trust informed staff about the research through an email invitation and staff meetings (in person and/or virtual) with Ulla. Patients were informed about the research through one or all of the following approaches, including (a) ‘poster’ on the clinic website and/or in the waiting room, visible in the process of booking an appointment, (b) a text message, targeted at people rebooking appointments who already had a diagnosis of a bacterial infection and (c) by staff reading out a short text or a ‘blurb’ to patients that were contacted by staff in person or over the phone. Potential participants could contact the authors directly or via an online virtual booking system. Ulla conducted in-person interviews with staff in two of the trusts, and staff were able to contact her to book interviews virtually and in person, and all patients’ interviews were virtual. The authors were responsible for fully informing potential participants about the research and obtaining informed consent to participate. Informed verbal or written consent was sought and secured for all interviews. At all but one trust, participants received a voucher worth £20 for their time. The remaining trust opted to receive bank cover for the time it took staff to partake in the research. Members of staff (*n* = 35) within four NHS trusts in England and patients (*n* = 7) from two of these trusts were interviewed. Research participants’ names have been replaced with pseudonyms, e.g., ‘Antonia’, to protect their identity. Interviews were primarily conducted by Ulla and lasted approximately one hour. They were professionally transcribed verbatim and subsequently uploaded and coded in NVivo 1.7 QSR International Pty Ltd. The interviews were semi-structured with topic guides to guide the conversation. The guides were amended several times over the course of the project, primarily to reflect the impact the pandemic had on the provision of sexual health care. Interviews with patients focused on their experience of care, including the diagnosis and treatment of sexually transmitted infection(s) during the pandemic. The interview this vignette draws on was conducted by Ulla on a virtual platform after Antonia booked an interview with her. Antonia described her race/ethnicity as ‘mixed white’ when asked during the conversation. After this, Antonia and Ulla discussed their experience of being women of colour and migrants in England and the differing ways in which racialised categories *were done* (St Louis 2022) in their countries of origin compared to the UK. Like this, Antonia said she was ‘white’ in her country of origin, but in the UK, she was not *quite white*. These experiences are an important part of this research, as Ulla argues elsewhere:

An integral and overlooked aspect of the researcher is the way in which she is racialised and her geographical location(s). As such, race and place are part of every aspect of a researcher’s research; the methods and methodological approaches she chooses, the interventions she observes, how she makes sense of them and the geographical spaces she observes them in and from; the way in which she is treated in these spaces and the way she imagines and communicates with her audience.… (McKnight 2022: 174).

Thus, during interviews and coding, Ulla paid attention to the ways in which race and geographical space came to matter in relation to experiences of care, diagnostic practices, and understandings of AMR and AMS. Consequently, during data analysis, we created the following code in NVivo: ‘negotiating racialised categories’. The significance of this code became apparent as Ulla conducted more interviews. Our analysis of Antonia’s interview focused on elucidating the vicissitudes of these concerns, specifically within the context of Antonia’s experiences with NHS care as a pregnant migrant woman of colour. An updated grounded theory approach guided data collection and thematic analysis of the data (Corbin and Strauss 1990; Mason 1996; Charmaz 2006). The project was supported by the National Institute for Health and Care Research (NIHR). It was given a favourable review by the London - Brighton and Sussex NHS Research Ethics Committee (IRAS 270126), the University of Sussex Sponsorship Sub-Committee, experts in social science, a lay review panel and patient representatives.

### Patient vignette and analysis

Antonia was 11 weeks pregnant when Ulla interviewed her. She was in her 30s and originally from a country in South America. Antonia went to a sexual health clinic in London after her partner had been diagnosed with *M. genitalium* at the same clinic, after having initially been treated there for a non-specific sexually transmitted infection (STI). During her appointment, Antonia relayed her partner’s history and disclosed that she had experienced post-coital bleeding and bouts of lower abdominal pain for some time and asked to be tested for *M. genitalium*. Her request was refused. Instead, she was offered a standard STI screening, and her symptoms were not treated. Her test results eventually all came back negative. Antonia’s experience of initially being denied a test for *M. genitalium* was extremely upsetting for her, considering her symptoms and her partner’s positive diagnosis. She was convinced that she had had an *M. genitalium* infection and that she should have received the same treatment as her partner. The refusal to screen Antonia for *M. genitalium* infection should be considered in light of the British Association for Sexual Health and HIV (BASHH) advice that testing is considered for people with post-coital bleeding (Soni et al., 2019: 941). They recommend testing and treating the current sexual partners of people infected with *M. genitalium* (Soni et al., 2019: 939) and treating them with the same antibiotic if they are positive - assuming resistance information does not indicate otherwise (Soni et al., 2019: 944). This raises the question of why Antonia was refused the appropriate test at a clinic that could provide it, the answer to which may relate in some part to the fact that these guidelines must be considered in light of background pressures *not* to test for *M. genitalium*, as mentioned previously. However, we are not suggesting that the Health Care Provider refused to test Antonia for *M. genitalium* for this reason. Indeed, the rationale behind their actions is not pertinent to this analysis. This is because the Health Care Provider’s logic was not made clear to Antonia, and our concern pertains to Antonia’s rendition of their actions and how this affected Antonia and her experience of care.

When Antonia’s partner’s symptoms returned over a month later, the couple went back to the same sexual health clinic together with separate same-day appointments. Antonia detailed her and her partners’ symptoms and sexual history to a Health Care Provider. She was offered a pregnancy test after explaining for the first time that she was trying to conceive and her period was late, but two home pregnancy tests had been negative. The Health Care Provider confirmed that Antonia was pregnant. At this point, Antonia was also tested for *M. genitalium*. The Health Care Provider caring for her explained that they would have to wait for the *M. genitalium* test results to be returned before Antonia could be offered treatment. Antonia assumed that some kinds of treatment might be more harmful during pregnancy, and she was reassured that the Health Care Provider seemed to be thinking about the pregnancy.

BASHH guidelines state that pregnant women considering treatment for *M. genitalium* should have a timely and informed discussion with their healthcare providers during which they discuss the risks associated with the medicines and *M. genitalium* in pregnancy (Soni et al., 2019: 944 - 5); where possible, treatment for *M. genitalium* should be delayed till after pregnancy (Soni et al., 2019: 944 - 5). However, if treatment is to proceed, BASHH advocates for a short course of Azithromycin for uncomplicated *M. genitalium* infections, as the antibiotic is “unlikely” to cause an increased risk of birth defects or adverse pregnancy outcomes.

When the test results eventually came back, they confirmed that Antonia had an *M. genitalium* infection. Antonia was prescribed ‘Minocycline 50 milligrams and 100-milligram tablets’ to be taken twice a day for four weeks. While Minocycline is contraindicated in pregnancy (NICE, 2022b), this advice is premised on documented effects of the antibiotic in (*non-human*) animals during the first trimester 1 (NICE, 2022b). The Health Care Provider she spoke with during the phone conversation explained that the medicine was safe for the ‘baby’ if taken before week 16. Antonia was told that she would only need to return to the clinic if she started experiencing symptoms again, but she was not told exactly what symptoms to look for. Prescribing Minocycline to Antonia is an example of the speculative work healthcare providers are forced to do when caring for pregnant people in the *absence* of data, weighing up the speculative costs versus the potential benefits of prescribing the drug despite it not being approved for use in pregnancy. Importantly, we are not suggesting that the decision to give Antonia Minocycline was wrong. Instead, we would like to draw out how appropriate and *good care* (Mol 2008) for individual patients involves this sort of individualised CBA that is forced to utilise inappropriate data and speculative variables. However, it is important to restate that it only became necessary for Antonia to take antibiotics antenatally because she was refused an *M. genitalium* test before she became pregnant. Thus, from Antonia’s perspective, any possible harm caused by taking antibiotics during pregnancy and/or not treating an *M. genitalium* infection during pregnancy would be a direct consequence of the refusal. Following this, the future effects of the refusal are potentially never-ending and unpredictable (e.g., if the baby is born with birth defects that *Antonia* attributes to her care).

We can only speculate on the logic that informed the Health Care Providers’ chosen treatment pathway and the circumstances under which the Health Care Providers provided care to Antonia. We do not, for example, know the particularities of Antonia’s diagnosis as it would be described by her healthcare providers - such as the resistance profile of her genotype or any of the institutional restrictions that might have influenced the way in which her sample was tested or the choices of care that were ultimately chosen. Furthermore, we do not know if the Health Care Providers that initially refused to test Antonia for *M. genitalium* would have made a different decision had Antonia disclosed that she was having unprotected sex with her partner because they were trying to conceive. Moreover, we do not know if the healthcare providers involved in Antonia’s care would agree with her rendition of the care she was provided with. These are important absences, and we cannot form a comprehensive picture of the logic that informed the care Antonia received without knowing more. However, our intention is not to paint the full picture. Indeed, we would argue that a full picture is impossible to paint. Thus, while we do not have enough information to conduct a thorough clinical CBA from the healthcare providers’ point of view, we suggest that the healthcare providers’ care in this instance might have taken into consideration the fact that Antonia desperately wanted antibiotic treatment. Yet, we would also like to point out that Antonia had not been (and could not be) fully informed of the potential risks of an *M. genitalium* infection or of taking Minocycline while pregnant.

During this phone conversation, the Health Care Provider suggested that Antonia might want to have an early pregnancy scan to make sure that the ‘baby was OK’ since she was still bleeding. The Health Care Provider told Antonia that she could try and phone herself to make an appointment. However, Antonia was not able to book a scan. None of the people she spoke with during her attempts had heard of *M. genitalium* or took her concerns seriously, including Antonia’s midwife, whom Antonia had only talked to on the phone at that point.

Antonia: I was like panicking, especially because I was trying to explain that the doctor asked me to have a scan to see. They were like, yeah, but you have…if you’re not bleeding you don’t have anything. But I said, yeah, but *I am bleeding*.

Ulla: So, they didn’t believe you that you had had bleeding?

Antonia: No. What they said to me was like, if you’re not bleeding *now*, you are okay.

About a week after trying to book a scan, Antonia received a call from the hospital saying she had an appointment. Antonia worried that she couldn’t properly ‘express’ herself, so she would always bring a note with her prescription and diagnosis written down to avoid misunderstandings. Even so, she felt that all the Health Care Providers she encountered outside of the sexual health clinic did not believe her and would not take her concern seriously. Having identified her distress, Ulla asked what Antonia felt should have been done differently for her to have felt cared for. She responded:

Antonia: So, I gave them the name, so I said what I was being treated for, I gave them the names of the medicine. So, on my understanding that’s fine, we don’t need to know everything about everything. But if they at least took the name of the disease that I gave to them, if they said, *okay, we’re going to look this thing to see if could have any impact, anything on the baby and we’re going to go back to you*, that could be make me feel more like, okay, they are trying to understand what’s going on with me. But they just said, no, you’re fine. Even the fact the bleeding can come back.

After a long day in the hospital, Antonia was told that her ‘baby’ was ‘fine’. She explained to Ulla that she felt okay now. However, these upsetting experiences, as well as Antonia and her partner’s initial appointments at the sexual health clinic, her positionality as a woman of colour migrant in England with English as an additional language and her desire to protect herself and her ‘baby’ significantly influenced her overall impression of the care she received.

## Discussion

Antonia’s’ experience of the diagnosis and treatment of *M. genitalium* was intricately influenced by practices beyond the remit of the sexual health clinic. In this way, Antonia judged the care she received not as separate moments of interaction between herself and separate healthcare providers practising care within the confines of their clinics and specific limitations therein - but as an assemblage that encompasses her various experiences with the NHS; interactions with the sexual health clinic, various healthcare providers, midwives, receptionists, appointments for early pregnancy scans etc., her desire to have a healthy baby and become a mother, concerns for her ‘unborn babies’ health, her experiences as a racialised migrant in the UK, her fear that having English as an additional language might have led to her being misunderstood, her understanding of antibiotics and STIs and the prenatal care she was provided with, initially being refused an *M. genitalium* test, her partner’s experiences, his treatment pathways and the tests and antibiotics that were prescribed and offered to him in addition to experiences and events not discussed during her interview with Ulla. Importantly, the assemblage materialises within a global context wherein ‘mixed-white’, ‘women of colour’, and ‘migrant women’ are made not to *matter* as much as cis white (British) women who are not from minority and/or racialised ethnic groups (Chambers et al., 2021; Nellums et al., 2021; Roberts 1999) (please see (Hollinshead et al., 2023) for a discussion of maternal health inequalities experienced by Gypsy, Roma and Traveller parents in the UK).

### Final thoughts

Antimicrobial Stewardship is not separate from care or Antonia’s experience. Her experience is not limited to the care she has received—it encompasses her partner’s care (the care people connected to her have received), their desire to become parents, their concern over the viability of the pregnancy and possible harm to the foetus, and Antonia’s expectations of care—which are informed by everything she has heard about care and knows about STIs and pregnancy. Furthermore, this framework materialises (Barad 2007) with the pervasive and pernicious belief that attributes the threat of infectious diseases to the geographical movement of racialised people (Wailoo 2020) migrants/asylum seekers while overlooking the movement of white European/North American travellers (Kamenshchikova et al., 2023; Kamenshchikova et al., 2018). These complexities and tensions relate to social and reproductive justice issues, and we must attend to them to avert the perpetuation of sexual and reproductive health inequalities that *M. genitalium* foretells. Like this Eaton and Stephens write:

Researchers and practitioners must acknowledge women’s health decisions, processes, and outcomes as extensions of their interactions with others and with systems, rather than as individual phenomena. The reproductive justice paradigm rises to this challenge, framing and analyzing reproductive health in terms of familial, community, societal, generational, political, and economic influences and their interactions. Reproductive justice recognizes that the ability of women and girls to make meaningful choices about their reproductive lives is shaped by intersecting systemic oppressions (e.g., racism, sexism, classism, heterosexism) (2020: 208-9).

Following this, we suggest that Antonia and patients like her will also make a cost-benefit analysis – based on what they know and experience- which has the potential to have an effect above and beyond the specific infection AMS was enacted to address. These *patient CBAs* should be given more attention and considered within a reproductive justice framework. In conclusion, we contend that we must attend to the ways in which “certain objects and practices – such as… race/ethnicity and migration from a geographical place [wherein disease is ontologically different] - come together in specific relational configurations” (McKnight 2022: 183) and have effect on the enactment of disease (Mol 2005; M’Charek 2013). This would enable a heightened acknowledgement and focus on how bodily autonomy and reproductive decision-making are embedded within broader social-structural frameworks (Eaton and Stephens 2020: 209), including immigration, race and access to NHS care, biomedical testing and diagnostic practices (McKnight 2022). Positing AMS as a reproductive justice concern would involve greater recognition and attention paid to the complexity and tensions in the decision-making around trade-offs and benefits among individual patients, their family members, the frontline staff they interact with and the geographical locations they may move between.

## Footnotes

2 In this article, we use the terms ‘cisgender woman’ or ‘woman’ when we are referring to (biomedical) research. However, when writing about the present, we adopt a language that is intentionally more non-binary/trans-inclusive (Ross and Solinger 2017a).

1 “Administration during the second or third trimester may cause discolouration of the child’s teeth and maternal hepatotoxicity has been reported with large parental doses.” (NICE, 2022b).
